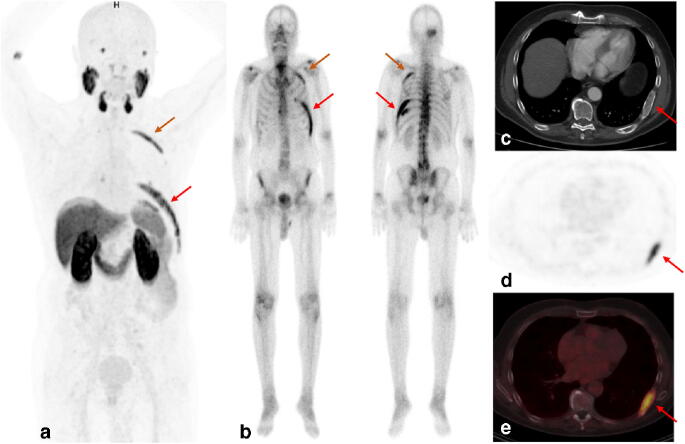# A rare case of polyostotic fibrous dysplasia detected on ^18^F-rhPSMA-7 PET/CT

**DOI:** 10.1007/s00259-020-04751-9

**Published:** 2020-04-16

**Authors:** Hui Wang, Matthias Eiber, Thomas Langbein

**Affiliations:** Department of Nuclear Medicine, Klinikum rechts der Isar, Technical University of Munich, School of Medicine, Ismaninger Straße 22, 81675 Munich, Germany

The prostate-specific membrane antigen (PSMA) has been proven to show high expression in prostate cancer cells [1]. The high binding affinity and internalization of PSMA radioligands makes it an excellent molecular target for theranostics of prostate cancer [1]. Positron emission tomography/computed tomography (PET/CT) using novel PSMA-targeting probes is increasingly used in recurrent and metastastic prostate cancer [2]. In addition, it is increasingly being used as an imaging modality for initial staging [3]. ^18^F-rhPSMA-7 is a new theranostic PSMA-targeting agent which allows radiolabeling with ^18^F and radiometals and is associated with minimal renal excretion [4]. However, physiologic and other pathologic forms of tracer uptake have to be considered carefully as potential pitfalls to image interpretation, such as ganglia, healing fracture, adrenal adenoma, primary lung cancer, and metastatic renal cell carcinoma [5].

Fibrous dysplasia is an uncommon skeletal disorder, accounting for 5 to 7% of all benign bone tumors [6]. Around 75% of cases have a monostotic form with only one bone involved, commonly the craniofacial bones, but the ribs, femur, and tibia may also be involved [7, 8]. PSMA-ligand uptake has been reported in monostotic (rib) fibrous dysplasia using ^68^Ga-PSMA-PET/CT, and this has to be differentiated from prostate cancer bone metastases [9]. Here, we present a case of uncommon polyostotic fibrous dysplasia identified on PSMA PET/CT imaging.

An 80-year-old man with histologically confirmed prostate cancer (Gleason score 7a, iPSA 4.26 ng/mL) by transurethral resection of the prostate (TUR-P) performed in February 2018 underwent ^18^F-rhPSMA-7 PET/CT imaging for staging in October 2018. No focal uptake in the prostate bed and no evidence of pelvic lymph node metastases was seen. However, intense tracer uptake was observed in the right parietal skull, in the left 3rd, 8th, and 9th ribs, and in the left ilium (red arrows in *a* MIP and *d* + *e* PET). The corresponding bone scan performed 2 weeks earlier had shown hypermetabolic lesions in the same areas (*b* bone scan). Finally, typical CT morphologic findings, including expansive changes with ground-glass appearance, cortical erosion, diffuse sclerosis, and well-circumscribed margins could be observed and, therefore, clearly confirmed radiologically polyostotic fibrous dysplasia (*c* CT) [10].

This case indicates that a thorough review of the CT dataset of a hybrid PSMA-ligand PET/CT is not only useful to correctly identify solitary or isolated lesions but also mandatory to avoid a potential pitfall and correctly differentiate extensive polyostotic fibrous dysplasia from metastatic bone lesions [11] when PSMA-ligand uptake in the bone is seen.